# Sex Steroid Hormone Single-Nucleotide Polymorphisms, Pesticide Use, and the Risk of Prostate Cancer: A Nested Case–Control Study within the Agricultural Health Study

**DOI:** 10.3389/fonc.2016.00237

**Published:** 2016-11-21

**Authors:** Carol H. Christensen, Kathryn Hughes Barry, Gabriella Andreotti, Michael C. R. Alavanja, Michael B. Cook, Scott P. Kelly, Laurie A. Burdett, Meredith Yeager, Laura E. Beane Freeman, Sonja I. Berndt, Stella Koutros

**Affiliations:** ^1^Office of Science, Center for Tobacco Products, Food and Drug Administration, Document Control Center, Silver Spring, MD, USA; ^2^Occupational and Environmental Epidemiology Branch, Department of Health and Human Services, Division of Cancer Epidemiology and Genetics, National Cancer Institute, National Institutes of Health, Rockville, MD, USA; ^3^Department of Epidemiology and Public Health, University of Maryland School of Medicine, Baltimore, MD, USA; ^4^Program in Oncology, University of Maryland Marlene and Stewart Greenebaum Comprehensive Cancer Center, Baltimore, MD, USA; ^5^Metabolic Epidemiology Branch, Department of Health and Human Services, Division of Cancer Epidemiology and Genetics, National Cancer Institute, National Institutes of Health, Rockville, MD, USA; ^6^Cancer Genomics Research Laboratory, Frederick National Laboratory for Cancer Research, Leidos Biomedical Research Inc., National Cancer Institute-Frederick, Frederick, MD, USA

**Keywords:** prostate cancer, pesticides, sex steroid hormones, single-nucleotide polymorphism, interaction

## Abstract

Experimental and epidemiologic investigations suggest that certain pesticides may alter sex steroid hormone synthesis, metabolism or regulation, and the risk of hormone-related cancers. Here, we evaluated whether single-nucleotide polymorphisms (SNPs) involved in hormone homeostasis alter the effect of pesticide exposure on prostate cancer risk. We evaluated pesticide–SNP interactions between 39 pesticides and SNPs with respect to prostate cancer among 776 cases and 1,444 controls nested in the Agricultural Health Study cohort. In these interactions, we included candidate SNPs involved in hormone synthesis, metabolism or regulation (*N* = 1,100), as well as SNPs associated with circulating sex steroid concentrations, as identified by genome-wide association studies (*N* = 17). Unconditional logistic regression was used to estimate odds ratios (ORs) and 95% confidence intervals (CIs). Multiplicative SNP–pesticide interactions were calculated using a likelihood ratio test. We translated *p*-values for interaction into *q*-values, which reflected the false discovery rate, to account for multiple comparisons. We observed a significant interaction, which was robust to multiple comparison testing, between the herbicide dicamba and rs8192166 in the testosterone metabolizing gene *SRD5A1* (*p*-interaction = 4.0 × 10^−5^; *q*-value = 0.03), such that men with two copies of the wild-type genotype CC had a reduced risk of prostate cancer associated with low use of dicamba (OR = 0.62 95% CI: 0.41, 0.93) and high use of dicamba (OR = 0.44, 95% CI: 0.29, 0.68), compared to those who reported no use of dicamba; in contrast, there was no significant association between dicamba and prostate cancer among those carrying one or two copies of the variant T allele at rs8192166. In addition, interactions between two organophosphate insecticides and SNPs related to estradiol metabolism were observed to result in an increased risk of prostate cancer. While replication is needed, these data suggest both agonistic and antagonistic effects on circulating hormones, due to the combination of exposure to pesticides and genetic susceptibility, may impact prostate cancer risk.

## Introduction

Farmers have a greater risk of prostate cancer than the general population or other occupational groups (1–3). Investigations within the Agricultural Health Study (AHS), a large prospective cohort of pesticide applicators, have identified links between prostate cancer, including aggressive forms of the disease, and pesticide exposure (4). Previous studies within the AHS have suggested that pesticides may interact with single-nucleotide polymorphisms (SNPs) along several different biological pathways to influence the risk of prostate cancer (5–9); however, additional biological pathways, including those involving hormones, have yet to be examined.

Prostate cancer has long been thought to be a hormonally modulated disease (10–12). Experimental and epidemiologic investigations suggest that certain pesticides may alter sex steroid hormone synthesis, metabolism and regulation, and thereby may interfere with sex steroid hormone homeostasis and alter risk of hormone-related cancers (13–18). Therefore, it is possible that variants in genes along this pathway may alter or amplify pesticide effects on hormone homeostasis and alter prostate cancer disease risk.

In the present hypothesis-generating study, we investigated genetic variation along the sex steroid hormone candidate pathway, as well as SNPs that have been associated with circulating sex steroid concentrations in genome-wide association studies (GWAS) as potential modifying factors of the relationship between pesticide exposure and prostate cancer risk.

## Materials and Methods

### Study Population and Genotyping

Criteria for selection into this case–control study have been described elsewhere (9). Briefly, this case–control study is nested within the AHS cohort, a prospective study that includes private and commercial pesticide applicators in Iowa and North Carolina (19). Cases were white male AHS pesticide applicators that were cancer free at enrollment, provided a buccal cell sample, and diagnosed with prostate cancer between enrollment (1993–1997) and 2004. Cancer incidence information, as well as tumor characteristics (Gleason score, stage) for the characterization of aggressive prostate cancer in the AHS (4), was obtained by linkage to cancer registry files in Iowa and North Carolina. Controls were white male AHS pesticide applicators frequency matched 2:1 to cases by age (±1 year). DNA was extracted from buccal cells using the Autopure protocol (Qiagen Inc., Valencia, CA, USA) at NCI’s Cancer Genomics Research Laboratory, where the genotyping was also performed. Genotyping analysis was conducted using the Custom Infinium^®^ BeadChip Assays (iSelect™) from Illumina Inc. and has been described elsewhere in detail (9). Exclusions due to quality control (insufficient/poor DNA quality or <90% completion rate for genotyping assays) or a genetic background that was inconsistent with European ancestry resulted in the final sample size of 776 cases and 1,444 controls. Study protocols were reviewed and approved by all relevant Institutional Review Boards.

### Exposure Information

Lifetime use of 50 specific pesticides was collected *via* self-administered questionnaires at enrollment in the AHS. Information was collected through both an initial enrollment questionnaire (including lifetime use of 22 pesticides) and supplemented with a take-home questionnaire (including lifetime use of an additional 28 pesticides). The take-home questionnaire was completed by 60.4 and 67.2% of the cases and controls, respectively. In a previous analysis, those who did and those who did not complete the take-home questionnaire were found to share similar characteristics, except for age (20).

Details of pesticide exposure assessment are presented elsewhere (21). The enrollment questionnaires asked participants to report the number of years that they personally applied each specific pesticide (1 year or less, 2–5, 6–10, 11–20, 21–30, or more than 30 years), as well as the number of days per year that they applied that pesticide (less than 5, 5–9, 10–19, 20–39, 40–59, 60–150, or more than 150 days). The number of years applied was multiplied by the number of days/year to construct a lifetime exposure days exposure metric for each pesticide. In addition, an intensity-weighted metric was calculated for each pesticide by multiplying the total lifetime days by an intensity score [intensity-weighted lifetime exposure days (IWLTED)]. The intensity score was derived from an algorithm based on several factors that may modify pesticide exposure, including mixing status, application method, equipment repair, and use of personal protective equipment (22, 23). This metric was subsequently categorized into three groups (none, low, and high IWLTED) for the present analysis using the median cut point of the data to divide low and high, based on the distribution of days among the controls for each of the pesticides included in the analysis. Data were obtained from AHS data release versions P1REL0712.04 and AHSREL201103.00.

Of the 50 pesticides evaluated at enrollment, we excluded pesticides with less than 10% prevalence among the controls in the present study due to insufficient numbers for analysis (trichlorfon, ziram, aluminum phosphide, ethylene dibromide, maneb/mancozeb, chlorothalonil, carbon tetrachloride/carbon disulfide, dieldrin, aldicarb, and 2,4,5-trichlorophenoxypropionic acid). We also constructed a single exposure variable for analysis of permethrin by combining information on crop and animal applications, which were asked about separately, leaving 39 pesticides available for analysis.

### Gene and SNP Selection

Genes and SNPs were selected using two approaches. In the first approach, we selected SNPs tagged for candidate genes involved in hormone biosynthesis, regulation, and/or metabolism. We identified hormone-related genes through a search of both the PubMed/Gene database and also the Kyoto Encyclopedia of Genes and Genomes (KEGG) catalog gene search tool using the terms “sex steroid hormone” or “hormone” or “testosterone” or “androgen” or “estrogen” in the keyword search. In addition, we sought information through a search of recent (last 5 years) topical literature reviews or original research studies evaluating the sex steroid hormone pathway to learn of common ways of identifying genes along this pathway (24–27). We excluded SNPs with a minor allele frequency less than 5% to allow for robust analysis of interactions between genetic loci and pesticide exposures. Following quality control exclusions, there were 1,100 SNPs within 56 hormone-related genes available for analysis.

In the second approach for SNP selection, we selected SNPs that were associated with circulating sex steroid hormone concentrations in GWAS (28–32). To identify these SNPs, we queried the GWAS Catalog^1^ for SNPs that influenced circulating steroid hormone concentration using the terms such as “androgens,” “estrogens,” “sex hormone-binding globulin (SHBG),” “testosterone,” and “estradiol.” We identified 17 SNPs at a threshold of *p* < 10^−5^ that were related to circulating hormone levels,^2^ of which 4 were genotyped on the iSelect platform. For the remaining SNPs (*n* = 13), we identified proxies *r*^2^ ≥ 0.80 where available using the online tool SNP Annotation and Proxy Search (SNAP)^3^ (33).

### Statistical Analysis

We used the SAS software (version 9.1; SAS Institute Inc., Cary, NC, USA) to estimate the association between pesticides and prostate cancer and stratified effects by genotype, as well as interaction *p*-values (*p-*interact), and PLINK (34) to estimate odds ratios (ORs) for SNP main effects. Unconditional logistic regression was used to estimate ORs and 95% confidence intervals (95% CIs) for the association between each SNP and the risk of prostate cancer and to estimate the statistical interaction between 39 pesticides and 1,100 steroid hormone pathway SNPs, as well as 17 SNPs or SNP proxies related to circulating hormone concentrations in GWAS studies, with incident prostate cancer. Genotypes were categorized by the number of variant alleles: 0, homozygous for wild-type; 1, heterozygous; and 2, homozygous variant. For the SNP main effect analysis, we assumed a log-additive genetic model (model as ordinal variable for number of variant alleles, 0, 1, or 2). For the interaction analysis, we assumed the dominant genetic model (i.e., modeling SNPs in two categories, zero copies of the variant allele, and one or two copies of the variant allele).

We examined the correlation between the 39 pesticides tested; in general, pesticides were not highly correlated with one another (pairwise Spearman correlation coefficients <0.40) (9). All models were adjusted for age (10-year categories) and study state (Iowa, NC, USA); other potential confounding variables, including smoking, body mass index (obesity), physical activity and a family history of prostate cancer, and use of correlated pesticide products, did not change the effect estimate significantly (<10% change in OR) and were not retained in the final models. Statistical interactions between pesticides and SNPs were assessed by including a cross-product term and evaluated using the likelihood ratio test, assuming a multiplicative model.

To adjust for the multiple statistical comparisons performed in this analysis, we translated *p*-values for interaction into *q*-values reflecting the false discovery rate (FDR) following the method by Benjamini and Hochberg (35). This method adjusts for the expected proportion of false discoveries as a function of the number of statistical tests performed. We performed FDR analyses by gene to account for the differing numbers of SNPs by gene, such that the number of comparisons for a given gene was 39 (for number of pesticides) multiplied by the number of tag SNPs for the gene. We performed FDR analysis separately for the two groups of selected SNPs, those tagged for sex steroid hormone pathway candidate genes and those that were associated with circulating hormone concentrations in GWAS.

## Results

Population characteristics of the prostate cancer nested case–control study have been previously published (5–9) and are also provided in Table 1. Cases and controls were similar with respect to age (matching factor). Prostate cancer cases and the cancer-free control group selected into this nested case–control study did not differ from the larger AHS cohort with respect to state of residence, pesticide applicator type, family history of prostate cancer, or disease characteristics for cases (i.e., stage and grade) (8, 36).

**Table 1:** **Selected characteristics of the nested case–control study of prostate cancer in the AHS**.

	Controls (*n* = 1,444)		Prostate cancer (*n* = 776)	
Selected characteristics	*n*	%	*n*	%
**Age at enrollment**				
<40	5	0.4	3	0.4
40–49	144	10.0	74	9.5
50–59	491	34.0	259	33.4
60–69	634	43.9	355	45.8
>70	170	11.8	85	11.0
**State of residence**				
Iowa	991	68.6	520	67.0
North Carolina	453	31.4	256	33.0
**Applicator type**				
Private	1,363	94.4	741	95.5
Commercial	81	5.6	35	4.5
**First degree family history**				
No	1,193	82.6	575	74.1
Yes	145	10.0	130	16.8
**Prostate cancer stage**				
I Local	–	–	578	74.5
II Regional	–	–	156	20.1
III Distant	–	–	12	1.5
IV Not staged	–	–	30	3.9
**Prostate cancer grade**				
Well differentiated	–	–	38	4.9
Moderately differentiated	–	–	547	70.5
Poorly differentiated	–	–	168	21.6
Undifferentiated	–	–	4	0.5
Not graded	–	–	19	2.4

The main effects of pesticide exposure on the risk of prostate cancer among nested case–control participants are displayed in Table S1 in Supplementary Material. There were no statistically significant positive associations between pesticides and prostate cancer in the nested case–control set. Evidence of significant positive associations has been observed between fonofos, terbufos, malathion, and aldrin and risk of prostate cancer in the larger cohort analyses (4). Tables 2 and 3, respectively, list the candidate genes along the hormone synthesis, metabolism, and regulatory pathway included in this study, and also the SNPs identified as related to circulating hormone concentrations in GWAS and their proxies included in the present study, where applicable. Table S2 in Supplementary Material reports the associations between SNPs tagged for sex steroid hormone pathway genes and risk of prostate cancer.

**Table 2:** **Candidate hormonal pathway genes examined for the interaction between 39 pesticides and risk of prostate cancer in the AHS**.

Genes in steroid hormone regulation, synthesis, and metabolism (# of SNPs)		
AKR1C1 (11)	CYP2E1 (21)	SST (9)
AKR1C2 (22)	CYP3A4 (6)	STAR (4)
AKR1C3 (26)	CYP3A43 (5)	SULF2 (13)
AKR1C4 (17)	CYP3A5 (7)	SULT1A1 (3)
AKR1D1 (19)	CYP3A7 (1)	SULT1A2 (3)
AR (6)	CYP7A1 (12)	SULT1B1 (10)
COMT (12)	CYP7B1 (19)	SULT1E1 (18)
CYP11A1 (15)	ESR1 (77)	SULT2A1 (13)
CYP11B1 (6)	ESR2 (33)	SULT2B1 (24)
CYP11B2 (8)	HSD11B1 (22)	UGT1A (all) (111)
CYP17A1 (14)	HSD17B1 (4)	UGT2A1 (29)
CYP19A1 (80)	HSD17B2 (51)	UGT2A3 (6)
CYP1A1 (5)	HSD17B3 (46)	UGT2B10 (5)
CYP1A2 (16)	HSD17B4 (29)	UGT2B11 (2)
CYP1B1 (33)	HSD3B1 (6)	UGT2B4 (18)
CYP24A1 (36)	HSD3B2 (14)	UGT2B7 (11)
CYP27B1 (6)	NCOA3 (11)	
CYP2B6 (12)	SHBG (8)	
CYP2C9 (15)	SRD5A1 (35)	
CYP2D6 (3)	SRD5A2 (23)	

**Table 3:** **Genome-wide association study (GWAS) SNPs related to circulating level of hormone**.

SNP (P if proxy)^a^^,^^b^	Original SNP (*r*^2^)	Region	Known gene/nearby gene	Reference	Hormonal relationship identified (GWAS *p*-value)^c^
rs334698 (P)	rs334699 (1.0)	1p31.3	None (nearby NFIA)	(37)	Testosterone (1.0 × 10^−41^)
rs334703 (P)	rs334699 (1.0)	1p31.3	None (nearby NFIA)	(37)	SHBG (2.0 × 10^−21^)
rs1260326 (P)	rs780093 (0.90)	2p23.3	None (nearby FNDC4, LOC729823, GCKR, and IFT172)	(28)	SHBG (9.0 × 10^−6^)
rs6900902 (P)	rs9322817 (1.0)	6q16.3	HACE1	(38)	Testosterone (6.0 × 10^−8^)
rs9332222 (P)	rs2185570 (1.0)	10q23.33	CYP2C9	(32)	SHBG (9.0 × 10^−6^)
rs4149056	n/a	12p12.1	SLCO1B1	(28)	SHBG (2.0 × 10^−08^)
rs727479	n/a	15q21.2	CYP19A1	(31)	Estradiol (3.3 × 10^−7^)
rs4784336 (P)	rs12596210 (0.93)	16q12	FTO	(31)	SHBG (9.0 × 10^−6^)
rs12600130 (P)	rs12596210 (0.85)	16q12.2	FTO	(31)	Testosterone (6.0 × 10^−8^)
rs1799941 (P)	rs12150660 (0.96)	17p13.1	SHBG	(30, 28)	SHBG (2.0 × 10^−106^)/testosterone levels (1 × 10^−41^)
rs1641536 (P)	rs1641537 (0.85)	17p13.1	SHBG	(28)	SHBG (2.0 × 10^−16^)
rs11552708 (P)	rs72829446 (0.93)	17p13.1	TNFSF13 and EIF4A1	(29)	DHT (9 × 10^−10^)
rs6259 (P)	rs72829446 (0.93)	17p13.1	SHBG	(29)	DHT (9.0 × 10^−10^)
rs2909430 (P)	rs1625895 (1.0)	17p13.1	TP53/reported gene SHBG	(28)	SHBG (2 × 10^−21^)
rs9901675	n/a	17p13.1	EIF4A1	(28)	SHBG (1.5 × 10^−07^)
rs727428	n/a	17p13.1	SHBG	(31)	SHBG (2.1 × 10^−16^)
rs1017993 (P)	rs2637125 (0.92)	19q13	SULT2A1	(32)	DHEAS (2.6 × 10^−19^)

*^a^SNP Annotation and Proxy Search (SNAP) (*r*^2^ > 0.80, CEU, 1,000 genome project, all defaults) (April 2014: https://www.broadinstitute.org/mpg/snap/ldsearch.php)*.

*^b^Statistical significance (SNP-trait *p*-value < 1.0 × 10^−5^) in the overall (initial GWAS + replication) population (http://www.genome.gov/27529028)*.

*^c^GWAS *p*-value (http://www.genome.gov/gwastudies/index.cfm?pageid=26525384#searchForm)*.

*SHBG, sex hormone-binding globulin; DHT, dihydrotestosterone; DHEAS, dehydroepiandrosterone*.

Tables 4 and 5 present the relation between herbicide and insecticide exposure, respectively, and prostate cancer risk by genotype at certain SNPs tagged for hormone pathway genes or SNPs associated with circulating hormone concentration in GWAS. Gene by pesticide interaction results are presented for associations that met all three of the following criteria: (1) significant interaction between the pesticide and the SNP with prostate cancer (uncorrected *p* for interaction <0.01), (2) significant association between the pesticide and prostate cancer (*p* < 0.05) in one genotype stratum with an accompanying monotonic exposure–response pattern, and (3) no association between the pesticide and prostate cancer in the other stratum. Results for statistical interactions that were qualitative in nature (i.e., interactions involving increased risk with exposure in one genotype group and decreased risk in the other) are not presented.

**Table 4:** **Interactions between herbicides and SNPs with prostate cancer risk (*p* < 0.01)**.

Exposure				None		Low exposure		High exposure			
SNP		Pesticide	Genotype	Ca/Co	REF	Ca/Co	OR (95% CI)	Ca/Co	OR (95% CI)	*p*-int^a^	*q*-value^b^
Hormonal pathway	Gene										
rs8192166	*SRD5A1*	Dicamba	CC	133/173	1.0	64/131	0.62 (0.41, 0.93)	49/142	0.44 (0.29, 0.68)	4.0 × 10^−5^	0.03
			CT + TT	189/396	1.0	108/229	0.95 (0.69, 1.30)	127/218	1.15 (0.85, 1.57)		
rs3798577	*ESR1*	Butylate	TT	162/256	1.0	10/42	0.40 (0.19, 0.83)	8/42	0.30 (0.14, 0.66)	8.0 × 10^−5^	0.12
			CT + CC	338/647	1.0	42/110	0.74 (0.50, 1.08)	64/97	1.28 (0.90, 1.80)		
Hormone GWAS	Affected hormone										
rs4784336	SHBG	Dicamba	AA	258/473	1.0	130/273	0.87 (0.65, 1.15)	152/274	1.00 (0.76, 1.32)	3.7 × 10^−3^	0.90
			AC + CC	66/98	1.0	42/87	0.65 (0.39, 1.08)	24/87	0.36 (0.20, 0.65)		
rs1017993	DHEAS	Alachlor	CC	182/406	1.0	135/282	1.07 (0.81, 1.41)	147/273	1.21 (0.92, 1.58)	9.2 × 10^−3^	1.00
			CT + TT	95/140	1.0	65/106	0.92 (0.61, 1.40)	47/113	0.60 (0.39, 0.93)		

*ORs adjusted for age and state*.

*^a^Strata with less than five observations per cell or qualitative interactions excluded. Interactions that were statistically significant (*p*-int < 0.01) and for which there was a significant association between the pesticide and SNP (*p* < 0.05) with a monotonic pattern in at least one genotype stratum are included in table*.

*^b^The *q*-value adjusts the *p*-value for multiple statistical comparisons using the false discovery rate (FDR) method*.

**Table 5:** **Interactions between insecticides and SNPs in hormonal candidate pathway with prostate cancer risk (*p* < 0.01)**.

				None		Low exposure		High exposure			
SNP^a^	Tagged gene	Pesticide	Genotype	Ca/Co	REF	Ca/Co	OR (95% CI)	Ca/Co	OR (95% CI)	*p*-int^b^	*q*-value^c^
rs384346	*HSD17B4*	Malathion	AA	150/308	1.0	114/229	1.04 (0.77, 1.40)	118/235	1.01 (0.75, 1.36)	2.0 × 10^−3^	0.16
			AT + TT	75/88	1.0	48/98	0.58 (0.36, 0.92)	34/93	0.43 (0.26, 0.71)		
rs384346	*HSD17B4*	Carbaryl	AA	241/466	1.0	79/162	0.91 (0.67, 1.25)	88/177	0.78 (0.55, 1.12)	2.8 × 10^−3^	0.16
			AT + TT	111/164	1.0	36/75	0.68 (0.42, 1.09)	14/62	0.27 (0.13, 0.55)		
rs7723390	*HSD17B4*	Terbufos	TT	335/644	1.0	112/197	1.11 (0.85, 1.46)	94/209	0.91 (0.69, 1.21)	2.1 × 10^−3^	0.16
			CT + CC	50/121	1.0	25/37	1.58 (0.85, 2.95)	32/32	2.47 (1.36, 4.51)		
rs7723390	*HSD17B4*	Fonofos	TT	418/796	1.0	68/129	1.04 (0.75, 1.44)	62/127	0.96 (0.69, 1.34)	5.5 × 10^−3^	0.20
			CT + CC	67/147	1.0	15/23	1.40 (0.68, 2.89)	25/21	2.51 (1.29, 4.90)		

*ORs adjusted for age and state*.

*^a^Pesticide–SNP interaction significant after false discovery rate adjustment. No pesticide–SNP interaction statistically significant after global FDR analysis*.

*^b^Strata with less than five observations per cell or qualitative interactions excluded. Interactions that were statistically significant (*p*-int < 0.01) and for which there was a significant association between the pesticide and SNP (*p* < 0.05) with a monotonic pattern in at least one genotype stratum are included in table*.

*^c^The *q*-value adjusts the *p*-value for multiple statistical comparisons using the false discovery rate (FDR) method*.

Among men with the homozygous wild-type genotype (CC) at SNP locus rs8192166 in the hormone-associated gene Steroid Reductase 5-alpha-1 (*SRD5A1*), we observed a reduced risk of prostate cancer among men who reported low use of dicamba (OR = 0.62, 95% CI: 0.41, 0.93) and high use of dicamba (OR = 0.44, 95% CI: 0.29, 0.68), as compared to those who reported no use of dicamba (Table 3). In contrast, there was no significant association between dicamba and prostate cancer among those carrying one or two copies of the variant T allele at rs8192166 (*p* for interaction = 4.0 × 10^−5^; *q*-value = 0.03) (Table 3). In addition, the association between dicamba use and prostate cancer was modified by genotype at rs4784336, located in an intronic region of the fat mass and obesity (*FTO*) gene. Although statistical power was limited, we repeated the same analyses, i.e., the two above listed interactions with dicamba and prostate cancer by genotype, restricting the analysis to more clinically significant subtype of prostate cancer and aggressive prostate cancer cases (distant stage, poorly differentiated grade, Gleason score of ≥7, or fatal prostate cancer), and we observed similar results (Table S3 Supplementary Material). We also reported statistical interactions (uncorrected *p*-interaction <0.01) between the herbicides butylate, dicamba, and alachlor with SNPs that were associated with circulating hormone concentrations in GWAS or tagged for hormone-related genes with prostate cancer risk, although these were not robust to correction for multiple statistical comparisons (*q*-value >0.05) (Table 3).

Table 4 presents insecticide associations with prostate cancer risk as modified by SNPs tagged for hormone pathway genes. We observed interactions between the insecticides terbufos, fonofos, malathion, carbaryl, and genetic variation in the estradiol metabolizing gene, *HSB17B4*, although these were not robust to adjustment for multiple comparisons (*q*-value >0.05). We did not observe statistically significant interactions or notable exposure–response trends between GWAS identified SNPs and insecticide exposure with prostate cancer. We also did not observe statistically significant interactions or notable exposure–response trends between fumigants or fungicides exposure and prostate cancer among those with certain measured genotypes (data not shown).

## Discussion

In this case–control study nested within the AHS cohort, we evaluated evidence of statistical interaction between pesticide exposure and SNPs in genes involved in steroid hormone signaling, metabolism, or regulation with the risk of prostate cancer. We observed several notable interactions which help to generate additional hypotheses as to how pesticides may interact with genetic variation in hormone-related genes to affect prostate cancer risk.

The interaction between dicamba and rs8192166 in *SRD5A1* and risk of prostate cancer remained significant after correction for multiple testing. We observed an inverse association between exposure to dicamba and prostate cancer risk among those carrying the homozygous wild-type genotype at a locus in this important hormone metabolism and regulatory gene. *SRD5A1* is one of the three steroid reductase 5A isoforms and is known to play a role in the bioconversion of testosterone to the more biologically active dihydrotestosterone (DHT) in the prostate gland (*SRD5A1* is highly expressed in prostate tissues) (39–41). DHT is involved in the transcription of androgen-response elements in the genome and facilitates cell proliferation and aspects of cell cycle control which may be aberrant in cancer cells. Dicamba has been shown to interact with hormone homeostasis in non-mammalian experimental systems at environmentally relevant levels (42). Among those with the homozygous wild-type genotype at rs8192166 in gene *SRD5A1*, dicamba may interact with the metabolic conversion to the more biologically potent androgen DHT, in turn potentially influencing or reducing the conversion of testosterone to DHT in the prostate. If so, decreased concentration of bioactive androgen in the prostate may reduce cell proliferation, thus reducing prostate cancer risk; however, there are no data to support this assertion. It may, however, help to explain previously observed risk estimates at and below unity for the association between this pesticide and prostate cancer in the larger AHS cohort (4).

We also observed evidence of a modifying role of SNPs that were previously associated with circulating hormone concentrations in GWAS, as well as several different statistical interactions between insecticides and two SNPs in the *HSD17B4* gene. These statistical interactions were not robust to multiple comparisons. Interestingly, we observed evidence of an apparent inverse association with prostate cancer with use of several pesticides in the presence of certain SNPs. There is evidence in the literature that shows an inverse association between some putative endocrine disrupting chemicals and hormonal cancers, although the mechanism by which these might decrease disease development is unclear. A small number of laboratory studies have tested members of the organophosphate and carbamate class of pesticides including carbaryl and reported significant inhibition of the metabolism of estradiol and testosterone in the presence of these pesticides (14, 17, 18, 43–45). Other studies in humans have shown a significantly reduced risk of testicular cancer (46) and a reduced risk of metastatic prostate cancer (47) associated with polychlorinated biphenyl exposure, known endocrine disrupting chemicals. Thus, a role for hormonal perturbation is plausible.

In our study, two organophosphate insecticides, terbufos and fonofos, were associated with an increased risk of prostate cancer among those carrying one or two copies of the variant allele in *HSD17B4* (rs7723390), which showed a low correlation with rs384346, and no association among men carrying two copies of the wild-type allele. Although there is a known role for *HSD17B4* in estradiol metabolism, laboratory studies have illustrated reduced metabolism of testosterone in the presence of fonofos (terbufos not tested) (18). Over the past decades, epidemiologic studies have suggested that the combined action of androgens and estrogens may play a role in prostate carcinogenesis and specifically in the development of aggressive prostate cancer (24, 41, 48). Notably, in the AHS, both terbufos and fonofos have been associated with the risk of aggressive prostate cancer (4). Thus, the observed increases and decreases in risk of prostate cancer for the insecticides in Table 4, which vary by *HSD17B4* SNP, may be explained by this complex balance of androgens and estrogens which have been shown to effect prostate cancer development.

There are several strengths and limitations to note. The potential for information bias is low in this study for several important reasons. Lifetime use of pesticides among this occupational cohort is measured with high validity and reliability (49, 50). Furthermore, given the quality of the exposure data and the large sample size, we were able to perform analyses at the individual pesticide level and not merely classes of chemical. There are likely few true confounding factors in the relation between genetic variability and disease. We were able to evaluate SNPs among hormone signaling, metabolism, and regulatory genes in this analysis comprehensively and used two methods to identify SNPs for inclusion into our study, i.e., tagging SNPs in candidate hormone pathway genes and GWAS identified SNPs. However, we were limited to the tagging SNPs for genes included in a group genotyping platform. We performed many different statistical tests in this evaluation, increasing the potential of identifying false-positive associations. However, we adjusted for this possibility using FDR methods. Despite the large sample size, we were still limited by the relatively small numbers of participants carrying the variant allele for rare SNPs, as well as small numbers for less frequently used pesticides such as fumigants or fungicides. Furthermore, we were underpowered to investigate whether there were notable interactions for aggressive prostate cancer (aggressive cases, *n* = 346), although much of the results presented were consistent if we only considered this subgroup of cases (Table S3 in Supplementary Material). Additionally, the AHS study participants included in this study are all white and occupationally exposed to pesticides, limiting the generalizability of our study results to the general population. However, our restriction to white men also limited the potential for population stratification to influence our findings.

High prior interest still remains in the hormonal pathway and its influence on prostate cancer initiation and progression, as well as the possible role of pesticides to disrupt this and other endocrine regulatory pathways. Future work should continue to consider newly identified SNPs that may affect circulating hormone concentrations, and further evaluation of risk of aggressive prostate cancer as cases accrue in this cohort. There is also a need for continued laboratory analyses to investigate the possible biological mechanisms through which pesticides may influence hormone synthesis, metabolism or regulation, and ultimately prostate cancer risk.

## Ethics Statement

The study was carried out in accordance with the recommendations of U.S. National Cancer Institute Special Studies IRB, the Westat Institutional Review Board, and the University of Iowa Institutional Review Board.

## Author Contributions

CC, KB, LF, SB, and SK contributed to the design, execution, and analysis of this paper. CC, KB, and SK drafted the manuscript. LB and MY were responsible for the genotyping performed. All the authors (including MC, SPK, GA, and MA) were involved in the critical revision of the manuscript.

## Disclaimer

Although the author is a FDA/CTP employee, this work was not done as part his/her official duties. This publication/presentation reflects the views of the author and should not be construed to reflect the FDA/CTP’s views or policies.

## Conflict of Interest Statement

The authors declare that the research was conducted in the absence of any commercial or financial relationships that could be construed as a potential conflict of interest.

## Footnotes

^1^http://www.ebi.ac.uk/gwas/home.

^2^http://www.ebi.ac.uk/gwas/docs/about.

^3^https://www.broadinstitute.org/mpg/snap/.

## Abbreviations

AHS, Agricultural Health Study; CI, confidence interval; FDR, false discovery rate; OR, odds ratio, PNCC, prostate nested case–control study; SNP, single-nucleotide polymorphism.
