# Supplementary material for: Sex Steroid Hormone Single-Nucleotide Polymorphisms, Pesticide Use, and the Risk of Prostate Cancer: A Nested Case–Control Study within the Agricultural Health Study
Source: Front Oncol. 2016 Nov 21;6:237. doi: 10.3389/fonc.2016.00237 (PMC5116569; doi:10.3389/fonc.2016.00237)
Supplement: Supplementary file 1 [file Table_1.PDF]

Supplemental Table 1: Odds ratios and 95% CI for the association between prostate cancer and 39 pesticides in the AHS PNCC

Lifetime exposure days	None		Low		High		P trend
	Ca/Co		*Ca/Co*	*OR* (95% CI)*	*Ca/Co*	*OR* (95% CI)*	
**Atrazine**	189/375	REF	277/494	1.13 (0.89, 1.42)	275/545	1.00 (0.79, 1.27)	0.512
**Dicamba**	324/573	REF	171/368	0.78 (0.61, 1.00)	181/360	0.82 (0.63, 1.05)	0.318
**Cyanazine**	391/698	REF	151/309	0.84 (0.65, 1.07)	141/305	0.78 (0.60, 1.00)	0.104
**Chlorimuron-ethyl**	487/955	REF	97/173	1.10 (0.83, 1.44)	47/106	0.83 (0.58, 1.20)	0.504
**Metolachlor**	369/712	REF	179/303	1.14 (0.91, 1.44)	133/303	0.84 (0.65, 1.07)	0.109
**EPTC**	530/1,063	REF	80/112	1.41 (1.03, 1.93)	63/129	0.95 (0.68, 1.31)	0.781
**Alachlor**	277/546	REF	223/400	1.08 (0.86, 1.35)	176/378	0.91 (0.72, 1.15)	0.298
**Metribuzin**	433/792	REF	83/183	0.84 (0.62, 1.12)	92/192	0.87 (0.66, 1.16)	0.351
**Paraquat**	592/1,082	REF	39/105	0.65 (0.44, 0.96)	34/67	0.82 (0.53, 1.29)	0.296
**Petroleum oil/Petroleum Distillate**	488/964	REF	56/113	1.00 (0.71, 1.41)	57/93	1.24 (0.87, 1.76)	0.236
**Pendimethalin**	474/856	REF	73/173	0.74 (0.55, 0.99)	79/165	0.85 (0.63, 1.14)	0.247
**Imazethapyr**	411/773	REF	143/232	1.16 (0.89, 1.50)	127/293	0.81 (0.62, 1.05)	0.119
**Glyphosate**	182/333	REF	285/561	0.90 (0.71, 1.14)	274/527	0.93 (0.73, 1.18)	0.886
**Butylate**	501/903	REF	43/122	0.64 (0.44, 0.93)	82/169	0.86 (0.64, 1.15)	0.305
**Trifluralin**	312/583	REF	186/362	0.95 (0.76, 1.20)	185/357	0.95 (0.75, 1.21)	0.762
**2,4-D**	135/218	REF	309/597	0.83 (0.64, 1.08)	288/582	0.79 (0.61, 1.04)	0.245
**2,4,5-T**	500/898	REF	93/158	1.07 (0.81, 1.42)	50/148	0.60 (0.42, 0.84)	0.004
**Permethrin (all)**	575/1,100	REF	78/117	1.24 (0.92, 1.69)	55/128	0.80 (0.57, 1.12)	0.224
**Terbufos**	406/803	REF	156/256	1.21 (0.95, 1.54)	124/246	1.01 (0.78, 1.30)	0.927
**Fonofos**	511/992	REF	104/181	1.12 (0.85, 1.47)	74/132	1.07 (0.78, 1.46)	0.605
**Lindane**	606/1,089	REF	38/94	0.69 (0.47, 1.03)	29/80	0.64 (0.41, 0.99)	0.031
**Carbofuran**	433/857	REF	129/212	1.18 (0.92, 1.51)	121/237	0.98 (0.77, 1.26)	0.845
**Chlorpyrifos**	451/854	REF	137/248	1.02 (0.80, 1.30)	164/313	0.98 (0.78, 1.23)	0.838
**Malathion**	225/399	REF	173/351	0.85 (0.67, 1.09)	142/310	0.80 (0.62, 1.04)	0.149
**Parathion**	627/1,176	REF	28/36	1.36 (0.82, 2.26)	24/50	0.83 (0.50, 1.38)	0.609
**Carbaryl**	352/633	REF	110/224	0.84 (0.64, 1.10)	107/256	0.62 (0.46, 0.84)	0.003
**Diazinon**	513/964	REF	68/117	1.03 (0.74, 1.42)	47/122	0.67 (0.47, 0.96)	0.032
**Phorate**	462/846	REF	79/155	0.73 (0.54, 0.99)	76/195	1.16 (0.92, 1.44)	0.041
**Aldrin**	481/896	REF	63/148	0.80 (0.57, 1.10)	83/165	0.93 (0.70, 1.26)	0.628
**Chlordane**	505/888	REF	77/190	0.69 (0.51, 0.92)	52/112	0.79 (0.55, 1.11)	0.092
**DDT**	373/699	REF	100/205	0.89 (0.68, 1.18)	104/239	0.77 (0.59, 1.01)	0.064
**Heptachlor**	545/1,003	REF	48/108	0.85 (0.59, 1.23)	52/124	0.77 (0.55, 1.10)	0.123
**Toxaphene**	585/1,084	REF	36/77	0.82 (0.54, 1.24)	43/86	0.87 (0.59, 1.28)	0.354
**Coumaphos**	610/1,144	REF	36/66	1.00 (0.66, 1.52)	30/66	0.83 (0.53, 1.30)	0.426
**DDVP**	603/1,123	REF	42/90	0.81 (0.55, 1.20)	44/93	0.85 (0.58, 1.24)	0.428
**Methyl bromide**	637/1,215	REF	52/108	0.88 (0.60, 1.28)	56/97	1.05 (0.71, 1.53)	0.799
**Benomyl**	662/1,242	REF	19/33	1.03 (0.58, 1.86)	17/36	0.84 (0.46, 1.52)	0.556
**Captan**	623/1,144	REF	30/68	0.81 (0.52, 1.27)	31/62	0.85 (0.54, 1.33)	0.491
**Metalaxyl**	590/1,113	REF	46/85	0.98 (0.67, 1.44)	36/67	0.92 (0.58, 1.44)	0.705

* Adjusted for age, race, and state.

Supplemental Table 3. Interactions between pesticides and SNPs (presented in Tables 3 and 4) and risk of aggressive* prostate cancer

**Exposure**			**None**			**Low Exposure**			**High Exposure**		**p-int**
**SNP**	**Pesticide**	**Genotype**	**Ca/Co**	**REF**		**Ca/Co**	**OR** (95% CI)**		**Ca/Co**	**OR** (95% CI)**	
rs8192166	Dicamba	CC	66/173	1.0		33/131	0.66 (0.39, 1.11)		20/142	0.38 (0.21, 0.68)	0.0002
		CT+TT	81/396	1.0		47/229	0.94 (0.61, 1.45)		57/218	1.20 (0.79, 1.82)	
rs3798577	Butylate	TT	70/256	1.0		6/42	0.49 (0.20, 1.21)		5/42	0.45 (0.17, 1.20)	0.019
		CT+CC	148/647	1.0		18/110	0.72 (0.42, 1.24)		31/97	1.43 (0.91, 2.23)	
rs4784336	Dicamba	AA	118/473	1.0		62/273	0.91 (0.63, 1.32)		70/274	1.03 (0.71, 1.49)	0.004
		AC+CC	30/98	1.0		18/87	0.59 (0.30, 1.18)		7/87	0.23 (0.09, 0.57)	
rs1017993	Alachlor	CC	92/406	1.0		55/282	1.32 (0.87, 1.99)		60/273	1.36 (0.9, 2.05)	0.470
		CT+TT	41/140	1.0		29/106	0.71 (0.38, 1.30)		24/113	0.50 (0.25, 0.98)	
rs384346	Malathion	AA	56/308	1.0		54/229	1.09 (0.73, 1.64)		59/235	0.87 (0.54, 1.39)	0.008
		AT+TT	31/88	1.0		24/98	0.70 (0.37, 1.32)		16/93	0.25 (0.09, 0.72)	
rs384346	Carbaryl	AA	104/466	1.0		40/162	1.21 (0.83, 1.75)		41/177	1.04 (0.72, 1.52)	0.006
		AT+TT	50/164	1.0		16/75	0.70 (0.37, 1.32)		5/62	0.25 (0.09, 0.72)	
rs7723390	Terbufos	TT	141/644	1.0		50/197	1.21 (0.83, 1.75)		46/209	1.04 (0.72, 1.52)	0.052
		CT+CC	25/121	1.0		11/37	1.37 (0.60, 3.12)		16/32	2.49 (1.17, 5.28)	
rs7723390	Fonofos	TT	171/796	1.0		31/129	1.16 (0.75, 1.81)		35/127	1.34 (0.87, 2.05)	0.022
		CT+CC	31/147	1.0		7/23	1.40 (0.54, 3.60)		16/21	3.53 (1.60, 7.81)	

* Aggressive prostate cancer defined as having one or more of the following tumor characteristics: distant stage, poorly differentiated grade, Gleason score of ≥7, or fatal prostate cancer (underlying cause, prostate cancer)

**ORs adjusted for age and state.
