# Supplementary material for: Sex Steroid Hormone Single-Nucleotide Polymorphisms, Pesticide Use, and the Risk of Prostate Cancer: A Nested Case–Control Study within the Agricultural Health Study
Source: Front Oncol. 2016 Nov 21;6:237. doi: 10.3389/fonc.2016.00237 (PMC5116569; doi:10.3389/fonc.2016.00237)
Supplement: Supplementary file 3 [file Table_3.PDF]

Supplemental Table 3. Interactions between pesticides and SNPs (presented in Tables 3 and 4) and risk of aggressive* prostate cancer

**Exposure**			**None**		**Low Exposure**		**High Exposure**		**p-int**
**SNP**	**Pesticide**	**Genotype**	**Ca/Co**	**REF**	**Ca/Co**	**OR** (95% CI)**	**Ca/Co**	**OR** (95% CI)**	
rs8192166	Dicamba	CC	66/173	1.0	33/131	0.66 (0.39, 1.11)	20/142	0.38 (0.21, 0.68)	0.0002
		CT+TT	81/396	1.0	47/229	0.94 (0.61, 1.45)	57/218	1.20 (0.79, 1.82)	
rs3798577	Butylate	TT	70/256	1.0	6/42	0.49 (0.20, 1.21)	5/42	0.45 (0.17, 1.20)	0.019
		CT+CC	148/647	1.0	18/110	0.72 (0.42, 1.24)	31/97	1.43 (0.91, 2.23)	
rs4784336	Dicamba	AA	118/473	1.0	62/273	0.91 (0.63, 1.32)	70/274	1.03 (0.71, 1.49)	0.004
		AC+CC	30/98	1.0	18/87	0.59 (0.30, 1.18)	7/87	0.23 (0.09, 0.57)	
rs1017993	Alachlor	CC	92/406	1.0	55/282	1.32 (0.87, 1.99)	60/273	1.36 (0.9, 2.05)	0.470
		CT+TT	41/140	1.0	29/106	0.71 (0.38, 1.30)	24/113	0.50 (0.25, 0.98)	
rs384346	Malathion	AA	56/308	1.0	54/229	1.09 (0.73, 1.64)	59/235	0.87 (0.54, 1.39)	0.008
		AT+TT	31/88	1.0	24/98	0.70 (0.37, 1.32)	16/93	0.25 (0.09, 0.72)	
rs384346	Carbaryl	AA	104/466	1.0	40/162	1.21 (0.83, 1.75)	41/177	1.04 (0.72, 1.52)	0.006
		AT+TT	50/164	1.0	16/75	0.70 (0.37, 1.32)	5/62	0.25 (0.09, 0.72)	
rs7723390	Terbufos	TT	141/644	1.0	50/197	1.21 (0.83, 1.75)	46/209	1.04 (0.72, 1.52)	0.052
		CT+CC	25/121	1.0	11/37	1.37 (0.60, 3.12)	16/32	2.49 (1.17, 5.28)	
rs7723390	Fonofos	TT	171/796	1.0	31/129	1.16 (0.75, 1.81)	35/127	1.34 (0.87, 2.05)	0.022
		CT+CC	31/147	1.0	7/23	1.40 (0.54, 3.60)	16/21	3.53 (1.60, 7.81)	

* Aggressive prostate cancer defined as having one or more of the following tumor characteristics: distant stage, poorly differentiated grade, Gleason score of ≥7, or fatal prostate cancer (underlying cause, prostate cancer)

**ORs adjusted for age and state.
